# Supplementary material for: Prevalence and predictors of clinically significant health anxiety among Saudi medical students: A multi-university cross-sectional study
Source: PLoS One. 2026 Apr 27;21(4):e0347337. doi: 10.1371/journal.pone.0347337 (PMC13120030; doi:10.1371/journal.pone.0347337)
Supplement: Attachment — Submitted filename: Point to point response.pdf [file pone.0347337.s006.pdf]

<u>Response to reviewer #1</u>

We sincerely thank the reviewer for their thoughtful and constructive comments. We appreciate the positive feedback regarding the improvements in the manuscript. We have carefully addressed all concerns, and the manuscript has been revised accordingly. Our detailed responses are provided below:

Comment 1:

Abnormal prevalence of past psychiatric history. Table 1 shows 450/650 participants (69.2%) reporting a personal psychiatric history, yet Figure 2 shows specific disorders at much lower rates (e.g., depression 16.3%). This discrepancy is unexplained and raises concerns about how this variable was defined and measured. Given that it yields an AOR of 3.74, its validity directly affects the regression model's interpretation.

Response:

We thank the reviewer for highlighting this important issue. The apparent discrepancy is due to differences in how these variables were collected. "Past psychiatric history" was assessed using a **mandatory binary (yes/no) item**, capturing any prior psychological or psychiatric condition, including both formally diagnosed and self-perceived conditions.

In contrast, the reporting of specific psychiatric conditions (Figure 2) was based on a **non-mandatory, multiple-response question**, allowing participants to optionally select specific disorders. Consequently, not all participants who reported a psychiatric history provided details on specific conditions, leading to lower frequencies for individual disorders.

To clarify this, we have:

- Added a detailed explanation in the **Methods section** describing how both variables were measured
- Included clarification in the **Results section** explaining the discrepancy
- Added a **limitation** in the Discussion acknowledging potential underreporting of specific conditions

Comment 2:

Inconsistent reporting of academic dissatisfaction as a predictor. The Abstract and Discussion describe academic dissatisfaction as a significant predictor of health anxiety; however, Table 1 shows p = 0.081 (non-significant), and it does not appear in the multivariable model (Table 3). This overclaiming must be corrected.

Response:
We appreciate the reviewer's careful observation. We agree that this was an overstatement.

We have revised the manuscript to ensure consistency:

- Removed academic dissatisfaction as a significant predictor from the Abstract
- Revised the **Discussion** to clarify that the association did not reach statistical significance and was not included in the multivariable model
- Reframed the interpretation to describe it as a **non-significant trend** rather than an independent predictor

Comment 3:

Misleading figure in the Abstract. The statement "higher prevalence was observed among females (49%)" appears to reflect the proportion of females among HA cases, not the prevalence of HA among females (which is 36.3% per Table 1). These are fundamentally different quantities and should be clearly distinguished.

Response:
We thank the reviewer for this important clarification. We agree that the previously reported value (49%) reflected the proportion of females among health anxiety cases, rather than the prevalence of health anxiety among females.

We have corrected this in the Abstract to accurately report prevalence:

- Health anxiety prevalence is now stated as 36.3% among females vs. 25.8% among males, consistent with Table 1.

Comment 4:

Missing Year 4 in Table 3. The multivariable regression includes Years 2, 3, 5, and 6 but omits Year 4 without explanation. Authors should clarify whether this was intentional and why.

Response:

We appreciate the reviewer for noting this lack of clarity. Year 4 was indeed included in the regression model but did not show a statistically significant association.

To address this, we have:

- Added a footnote to Table 3 stating:
  "Year 1 was used as the reference category. All academic years were included in the model; Year 4 is not shown as it was not statistically significant."
- Included a brief clarification in the Results section

Comment 5:

Figure 1 p-value notation. "p = 0.000" should be corrected to "p < 0.001" per standard statistical reporting conventions.

Response:

We thank the reviewer for this important correction. In response, we have removed the p-value notation ("p = 0.000") from within the figure itself and instead reported it correctly in the figure footnote as **"p < 0.001"**, in accordance with standard statistical reporting conventions. This change improves both clarity and adherence to reporting guidelines.
