# Supplementary material for: Prevalence and predictors of clinically significant health anxiety among Saudi medical students: A multi-university cross-sectional study
Source: PLoS One. 2026 Apr 27;21(4):e0347337. doi: 10.1371/journal.pone.0347337 (PMC13120030; doi:10.1371/journal.pone.0347337)
Supplement: Attachment — Submitted filename: Table 1.docx [file pone.0347337.s001.docx]

**Table 1: Demographic characteristics and prevalence of health anxiety among participants**

**Characteristic**

**Health Anxiety**

**P-value2 No**

**N = 4551**

**Yes**

**N = 1951**

**Age 23 (22-24) 23 (21-25) 0.04*****

**Academic Level**

**<0.001*****

**Year 1 54 (12%) 21 (11%)**

**Year 2 50 (11%) 47 (24%)**

**Year 3 43 (9.5%) 34 (17%)**

**Year 4 82 (18%) 34 (17%)**

**Year 5 139 (31%) 54 (28%)**

**Year 6 87 (19%) 5 (2.6%)**

**Gender**

**Female 167 (37%) 95 (49%) 0.004*****

**Male 288 (63%) 100 (51%)**

**Residence**

**Rural (Countryside) 91 (20%) 69 (35%) <0.001*****

**Urban (City) 364 (80%) 126 (65%)**

**University Type**

**Govermental University 421 (93%) 125 (64%) <0.001*****

**Private University 34 (7.5%) 70 (36%)**

**Satisfied with academic performance**

**No, I am not satisfied. 172 (38%) 88 (45%) 0.081**

**Yes, I am satisfied. 283 (62%) 107 (55%)**

**Family member in the medical field**

**No, I don't. 201 (43%) 86 (44%) 0.9**

**Yes, I do. 254 (57%) 109 (56%)**

**Respected Authors**

**The reviewer needs clarification**

**Should your table be like this**

**Male Female Total**

**388	262	650**

**Anxiety	no anxiety	total**

**Female	167(63.7%)	95 (36%)	262**

**Male	288 (74.22%)	100(25.77%)	388**

**Accordingly correct Rural, Urban, University Type, Govt, Private and other tables and interpretation.**

**Please correct me or let me know your arguments in this respect.**
