# Supplementary material for: Prevalence and predictors of clinically significant health anxiety among Saudi medical students: A multi-university cross-sectional study
Source: PLoS One. 2026 Apr 27;21(4):e0347337. doi: 10.1371/journal.pone.0347337 (PMC13120030; doi:10.1371/journal.pone.0347337)
Supplement: Attachment — Submitted filename: Response to Reviewers.docx [file pone.0347337.s002.docx]

**Point-by-point response**

**Reviewers comments:**

| **Comment** | **Reply** |
| --- | --- |
| **Reviewer #1:** | |
| **1. Sample Size Inconsistency and study flow** There is a concerning discrepancy in the reported sample sizes throughout the manuscript. The abstract and methods section state 650 participants, while page 11 mentions 620 students completed the questionnaire. Please clarify the final sample size and provide a detailed account of participant flow. I recommend including a CONSORT-style flow diagram showing the number of students initially approached, those who consented, completed the survey, and any exclusions. This transparency is essential for readers to assess the study's representativeness and potential selection bias. | Thank you for highlighting this discrepancy. You are correct—the final sample size was 650 students, as reported in the Abstract and Results. The Methods section mistakenly stated 620, and this has now been corrected for consistency. Regarding the suggestion of a CONSORT-style flow diagram, we appreciate the recommendation; however, as this is a cross-sectional survey rather than a clinical trial, a CONSORT diagram is not applicable. Instead, we have clarified the participant recruitment and completion process in the Methods to ensure transparency. |
| **2. Network analysis methodology requires expansion** While the network analysis of disease fears is innovative and potentially valuable, the methodology section lacks sufficient detail for replication. Please provide comprehensive information, including: (a) the specific algorithm used for network construction, (b) statistical thresholds for determining edge weights and connections, (c) measures used to assess network properties (betweenness centrality calculations), and (d) validation procedures for the network structure. Additionally, the interpretation of "scale-free properties" and "betweenness centrality = 0.42" requires more explanation for readers unfamiliar with network analysis. | Thank you for this insightful comment. We agree that more detail is required to allow replication of the network analysis. We have now expanded the Methodology section to include a clear description of the algorithm used for network construction, the statistical thresholds applied for edge weights, and the measures adopted to assess network properties (including calculation of betweenness centrality). We have also added a description of the validation procedures undertaken to ensure robustness of the network structure. |
| **3. Effect sizes and clinical significance** The manuscript focuses heavily on statistical significance while giving limited attention to effect sizes and clinical meaningfulness. For instance, the correlation between health anxiety and quality of life (r = -0.25) is statistically significant but represents a small-to-moderate effect. Please discuss what this correlation means in practical terms for student wellbeing and daily functioning. Similarly, interpret the clinical significance of other key findings, such as the odds ratios, in terms of real-world impact on medical education and student support needs. | Thank you for this valuable suggestion. We agree that reporting statistical significance alone is insufficient without addressing the magnitude and clinical relevance of the findings. We have therefore expanded the Discussion to interpret effect sizes and odds ratios in practical terms. |
| **4. Private university finding requires deeper analysis** The six-fold increased odds of health anxiety among private university students (AOR = 6.14, 95% CI: 3.66–10.5) is the most striking finding in your study, yet it receives insufficient discussion. This dramatic difference suggests important underlying factors that warrant exploration. Please provide more detailed analysis of potential explanations, such as differences in: academic pressure and expectations, financial burden and family investment, student support services availability, class sizes and faculty-student ratios, or institutional culture and competitive environment. This finding has significant implications for medical education policy and deserves more thorough examination. | Thank you for underscoring this point. We agree that the markedly higher odds of health anxiety among private-university students warrants deeper consideration. We have substantially expanded the Discussion to explore plausible explanations (academic pressure and assessment practices, financial burden and family investment, availability and accessibility of student support services, class sizes/faculty–student ratios, and institutional culture/competitiveness), while emphasizing that our cross-sectional design precludes causal inference and that unmeasured confounding may remain. We also outline policy implications for targeted screening and support within private institutions. |
| **5. Temporal and academic calendar considerations** The study period spanning November 2024 to April 2025 encompasses different academic phases, examination periods, and potentially varying stress levels throughout the academic year. Please address how the timing of data collection might have influenced results. Consider discussing: (a) whether data collection coincided with examination periods or clinical rotations, (b) potential seasonal effects on mental health, (c) how different academic years might have been at different stages of their curriculum during data collection, and (d) whether this temporal variation was controlled for or represents a limitation that could affect interpretation of year-level differences. | Thank you for highlighting this important point. We agree that the timing of data collection may have influenced our results. The survey period (November 2024–April 2025) spanned different academic phases, including coursework, clinical rotations, and examination periods, which are likely to affect student stress and anxiety levels. Seasonal variation in mental health may also have contributed. While we did not control for the exact timing of responses in relation to academic calendar events, we recognize that this temporal variation could partly explain observed differences between year levels. We have therefore added this as a limitation in the Discussion, noting that fluctuations in academic stress across the academic year may have influenced the prevalence estimates and associations observed. |
| **6. Multiple comparisons and statistical rigor** Given the numerous statistical tests performed across multiple demographic and academic variables, please consider implementing appropriate corrections for multiple comparisons or discuss why such corrections were deemed unnecessary. This would strengthen the statistical rigor and help distinguish between truly significant associations and those that might have emerged by chance. | Thank you for this important observation. We recognize the risk of type I error when conducting multiple statistical tests. Given that our analyses were exploratory in nature and aimed to identify potential associations rather than establish definitive causal relationships, we did not apply formal corrections for multiple comparisons. Instead, we emphasized effect sizes, confidence intervals, and consistency with prior literature to guide interpretation. Nevertheless, we have now acknowledged in the Discussion that some findings may have arisen by chance due to multiple testing, and we recommend cautious interpretation of marginally significant results. |
| **7. Cultural context elaboration** While you mention cultural factors, the discussion of how Saudi cultural context might influence health anxiety expression, reporting, and management is underdeveloped. Please expand on how cultural attitudes toward mental health, gender roles, family expectations, and healthcare seeking might have influenced your findings. | Thank you for this valuable suggestion. We agree that the Saudi cultural context plays an important role in shaping the expression, reporting, and management of health anxiety. We have expanded the Discussion to consider how cultural attitudes toward mental health, gender roles, family expectations, and healthcare-seeking behaviors may have influenced both prevalence patterns and help-seeking tendencies in our sample. |
| **Reviewer #2:** | |
| This cross-sectional study investigates health anxiety and its relationship with quality of life among Saudi medical students, a subject often overlooked. By using established tools like the SHAI and SF-12 it provides quantitative data and insights from a specific geographic and cultural context. The findings are useful for developing targeted mental health support in medical education, ultimately enhancing student wellness and potentially improving future patient care. Its network analysis also offers novel insights into anxiety-related fears. However, there are significant concerns as outlined below, and revisions to the manuscript are deemed necessary. | |
| 1. In the Abstract, it states that health anxiety was observed in many females (49%), yet the adjusted odds ratio is reported as low at 0.430, which seems contradictory. Upon reviewing the main text, the correct adjusted odds ratio of 0.430 actually applies to males. | Thank you for pointing this out. You are correct—the adjusted odds ratio of 0.430 applies to males, not females. The wording in the Abstract was misleading, and we have now corrected it to ensure consistency with the main text. |
| 2. The selection of subjects for this study may be subject to selection bias. Since the online questionnaire was distributed via social media and university channels, students with a higher interest in their own health and mental well-being were more likely to respond. Consequently, the prevalence of health anxiety may be overestimated. | Thank you for this important observation. We agree that the use of an online questionnaire distributed through social media and university channels may have introduced selection bias, as students with a stronger interest in health and mental well-being could have been more likely to participate. We have acknowledged this limitation in the Discussion section and clarified that the prevalence of health anxiety in our sample may therefore be overestimated. |
| **Reviewer #3:** | |
| The reviewer needs clarification Should your table be like this Male Female Total 388 262 650 Anxiety no anxiety total Female 167(63.7%) 95 (36%) 262 Male 288 (74.22%) 100(25.77%) 388 Accordingly, correct the Rural, Urban, University Type, Govt, Private, and other tables and interpretations. Please correct me or let me know your arguments in this respect. The tables look incorrect to me as percentages are not calculated in the correct manner, which may influence data analysis and interpretation. However, if corrected, things can fall in line. | We thank you for your thoughtful comment and for carefully reviewing our tables. In the earlier version, we presented the data using column percentages, which is a valid approach and not incorrect statistically. However, we agree with your observation that presenting the results as row percentages (as in your suggested format) provides clearer interpretation of the distribution of health anxiety within each subgroup. Accordingly, we have revised Table 1 to present both counts and row percentages, and we added a Total column for clarity. We believe this new format is easier to understand and aligns with your recommendation, while not altering the underlying data or study conclusions. |
| **Reviewer #4:** | |
| Dear colleagues, It was a pleasure to read your paper. It is my opinion that the manuscript articulates a clear thesis: to estimate the prevalence of clinically significant health anxiety among Saudi medical students, identify its predictors, and examine its impact on quality of life. This is well-stated in the title, abstract, and introduction. The topic is relevant and timely, given the growing recognition of mental health issues in medical education. Some areas of improvement that I can recommend: | |
| 1. The introduction could be sharpened slightly. Decreasing some redundancies in the epidemiological descriptions can improve flow without losing critical context. | Thank you for this constructive feedback. We agree that the introduction contained some redundancies, particularly in the epidemiological descriptions. We have now streamlined this section by reducing overlapping prevalence details while retaining the essential context needed to frame the study. This has improved the flow and readability of the introduction without compromising comprehensiveness |
| 2. Some newer references on mental health interventions in medical education could enrich the context. | Thank you for this helpful suggestion. We agree that incorporating newer references on mental health interventions in medical education would strengthen the context. We have now added recent studies. |
| 3. Discussing the limitations of convenience sampling and cross-sectional design earlier in the methodology rather than only in the discussion could improve transparency. More discussion on potential biases introduced by online data collection would help. | Thank you for this valuable suggestion. We agree that greater transparency regarding the limitations of our study design is important. We have now added a statement in the Methodology section acknowledging the inherent limitations of using convenience sampling and a cross-sectional design. In addition, we have expanded the Discussion to further elaborate on the potential biases introduced by online data collection and how these may have influenced our findings |
| 4. Also, information about the response rate and the handling of missing data should be clarified. | Thank you for raising this point. Since the study was conducted using an online questionnaire (Google Forms), calculating a conventional response rate was not possible. To minimize missing data, all questions were set as mandatory, and therefore the dataset used for analysis was complete without missing values. |
