# Supplementary material for: Prevalence and predictors of clinically significant health anxiety among Saudi medical students: A multi-university cross-sectional study
Source: PLoS One. 2026 Apr 27;21(4):e0347337. doi: 10.1371/journal.pone.0347337 (PMC13120030; doi:10.1371/journal.pone.0347337)
Supplement: Attachment — Submitted filename: Reviewer_comments_auresp_2.docx [file pone.0347337.s003.docx]

We would like to sincerely thank the reviewers and the editor for their valuable time, thoughtful comments, and constructive suggestions that have significantly improved the quality and clarity of our manuscript. We appreciate the careful consideration given to our work throughout the review process. We are pleased to note that all reviewers’ previous comments have been fully addressed in this revised version. Below, we provide detailed responses to the remaining points raised and outline the specific revisions made to further strengthen the manuscript.

**Reviewer #1:**

**Comment 1:**

Reporting of Odds Ratio in Abstract
The statement "male gender (AOR = 0.430, 95% CI: 0.276–0.664)" in the Abstract is confusing and may mislead readers into thinking males have higher risk. Please revise to clearly indicate that females have elevated odds. Consider rephrasing as: "Female students demonstrated 2.3-fold higher odds of health anxiety compared to males (AOR for males = 0.430, 95% CI: 0.276–0.664)" or report the odds ratio for females directly (AOR = 2.33, 95% CI: 1.51–3.62).

**Response:**

We thank the reviewer for this insightful comment. We agree that the phrasing in the Abstract may lead to misinterpretation. Accordingly, we have revised the statement to clearly indicate that female students had higher odds of health anxiety. The revised sentence now reads: “Female students demonstrated 2.3-fold higher odds of health anxiety compared to males (AOR = 2.33, 95% CI: 1.51–3.62).”This change improves clarity and ensures that the findings are accurately interpreted.

**Comment 2:**

Insufficient Discussion of Private University Finding
The six-fold increased odds of health anxiety among private university students (AOR = 6.14) represents the most striking finding in your study, yet the discussion remains inadequate despite revisions. Please provide a more comprehensive analysis including: (1) specific structural differences between public and private medical schools in Saudi Arabia (e.g., tuition costs, admission criteria, faculty-student ratios); (2) whether religious or cultural factors may contribute to these differences; and (3) concrete policy implications for private institutions. Consider adding a separate dedicated paragraph or a supplementary table comparing baseline characteristics between public and private university students.

**Response:**

We thank the reviewer for this important suggestion. We agree that the six-fold increased odds of health anxiety among students from private universities (AOR = 6.14) deserves a fuller discussion. In response, we have expanded the Discussion (new paragraph) to address likely structural, cultural, and financial contributors. We also included concrete policy implications for private medical colleges. These additions are intended to help readers interpret this striking finding and guide institutional responses**.**

**Comment 3:**

Statistical Methodology Issues
**Issue 3.1:** While your response to reviewers appropriately explains the rationale for not applying multiple comparison corrections, this important methodological decision should be explicitly stated in the Methods section or Limitations, not only in the rebuttal letter. Please add: "Given the exploratory nature of this study, no formal correction for multiple comparisons was applied. We relied on effect sizes, confidence intervals, and consistency with prior literature to guide interpretation."

**Issue 3.2:** The manuscript lacks model fit statistics for the logistic regression. Please report goodness-of-fit indicators such as Hosmer-Lemeshow test results, Area Under the Curve (AUC), pseudo-R², or AIC values to allow readers to evaluate model adequacy.

**Response:**

3.1. We thank the reviewer for highlighting this point. We agree that our rationale regarding multiple comparison correction should be transparently stated in the manuscript. We have now added the sentence to the Methods section (and repeated it briefly in the Limitations for clarity).

3.2. We appreciate the reviewer’s valuable suggestion. We have now included model fit and discrimination statistics in the Results section to improve transparency and assess model adequacy. Specifically, we report the Hosmer–Lemeshow test, Nagelkerke pseudo-R², and the Area Under the Curve (AUC).

**Comment 4:**

Methodological Transparency
**Issue 4.1:** While you indicate that calculating a conventional response rate was not possible, you should still provide information about the sampling frame. Specifically, state: (1) the number of universities contacted, (2) the estimated total population of medical students across these institutions, and (3) approximate distribution channel reach. This information is essential for assessing representativeness and potential selection bias.

**Issue 4.2:** Although temporal variation in data collection is now mentioned in Limitations, the description should be more specific. Please explicitly state which academic phases coincided with your survey period (e.g., "Data collection occurred during midterm examinations [November-December], clinical rotation transitions [January], and final exam preparation [March-April]"). Additionally, acknowledge that this timing may have systematically inflated anxiety prevalence estimates and recommend that future studies control for academic calendar phase.

**Response:**

4.1. We thank the reviewer for this helpful suggestion. We have now added a clear description of the sampling frame to enhance methodological transparency and allow readers to assess representativeness. The revised text in the Methods section provides the number of universities contacted, the estimated total population of medical students across these institutions, and the approximate distribution reach of our survey link.

4.2. We appreciate the reviewer’s suggestion to make the timing of data collection more specific. We have now clarified the exact academic phases that coincided with data collection and acknowledged their potential influence on anxiety levels. The revised sentence has been added to the Limitations section.
