# Supplementary material for: Prevalence and predictors of clinically significant health anxiety among Saudi medical students: A multi-university cross-sectional study
Source: PLoS One. 2026 Apr 27;21(4):e0347337. doi: 10.1371/journal.pone.0347337 (PMC13120030; doi:10.1371/journal.pone.0347337)
Supplement: Attachment — Submitted filename: Point to point response.docx [file pone.0347337.s005.docx]

**Reviewer #1 comments**

**Major Comment 1:** Insufficient Depth in Private University Discussion

While the authors have expanded the discussion of the six-fold increased odds of health anxiety among private university students (AOR = 6.14), this remains the most striking finding and warrants more substantive analysis. The current discussion mentions structural differences (tuition, admission criteria, faculty ratios) and cultural factors only in general terms without providing concrete comparative data or context. We recommend that the authors either add a supplementary table comparing specific characteristics between public and private institutions (e.g., actual tuition costs in Saudi Riyals, faculty-to-student ratios, number of counselors per campus, scholarship rates) or incorporate these quantitative details directly into the discussion text. Additionally, the dismissal of cultural and religious factors as having "limited evidence" is premature; even without direct empirical data from this study, the authors should propose plausible hypotheses about how institutional culture, family expectations, religious practices, or peer support networks might differ between institution types and influence health anxiety expression or reporting. We suggest revising the cultural factors paragraph to articulate specific mechanisms and recommend concrete directions for future mixed-methods research rather than simply acknowledging the gap. This enhanced discussion would help readers interpret this striking OR and guide institutional responses appropriately.

**Response:**

Thank you for this insightful comment. We agree that the markedly elevated odds of health anxiety among private university students (AOR = 6.14) is one of the most important findings of the study and warrants deeper interpretation. Accordingly, we substantially expanded the Discussion to provide a more comprehensive framework for understanding this association. Specifically, we elaborated on plausible institutional mechanisms such as tuition-related financial pressure, differences in academic structure and competitiveness, and variability in the availability of student support services. In addition, we revised the paragraph on cultural and religious factors to avoid prematurely dismissing their potential role. Instead, we proposed specific, testable hypotheses regarding how institutional culture, family expectations, peer norms, stigma, and spiritual coping practices may influence the expression and reporting of health anxiety. Finally, we added clear recommendations for future mixed-methods research (quantitative institutional comparisons complemented by qualitative interviews) to better characterize these institutional and sociocultural pathways.

**Major Comment 2:** Policy Recommendations Lack Specificity and Actionability

The policy recommendations paragraph provides valuable general suggestions but lacks the concrete, evidence-based specificity needed for institutional implementation given your strong empirical findings (6.14-fold odds for private universities, 3.25-fold for Year 2 students, 2.33-fold for females). We recommend revising each recommendation to include actionable details: (1) for financial support, specify mechanisms such as automatic tuition deferrals for students screening positive for mental health concerns or need-based emergency grants with clear eligibility criteria; (2) for mental health services, propose specific standards such as minimum counselor-to-student ratios (e.g., 1:500 based on international guidelines), same-day walk-in availability, and measurable wait-time targets; (3) for academic support, describe year-specific interventions such as mandatory peer mentoring for Year 2 students (your highest-risk group) or curriculum modifications providing earlier clinical context; (4) for screening programs, recommend validated instruments (e.g., SHAI as used in your study), specific timing aligned with high-stress periods identified in your limitations (November-December exams, March-April finals), and systematic follow-up protocols for high-risk subgroups (females, rural residents, those with psychiatric history). Adding implementation timelines, citing comparable successful models from the literature where available, and suggesting evaluation metrics would transform these recommendations from aspirational statements into a practical roadmap for Saudi medical institutions seeking to address health anxiety among their students.

**Response:**

We sincerely thank the reviewer for this valuable recommendation. We agree that policy recommendations should be directly linked to our highest-risk groups and written in an implementable, action-oriented format. We therefore revised the policy recommendations section to include specific, evidence-informed actions tailored to our strongest empirical findings (private universities, Year 2 students, and female students). The revised text now outlines actionable strategies, including structured financial support mechanisms (e.g., emergency aid and tuition support options), minimum standards for mental health service accessibility (including staffing benchmarks and measurable wait-time targets), year-specific academic interventions (such as structured peer/near-peer mentoring for Year 2 students), and screening protocols using validated instruments (SHAI) aligned with high-stress academic periods identified in our limitations. We also added suggested evaluation metrics to support institutional monitoring of implementation outcomes.

**Reviewer #5: Reviewer comments**

Major Comments

**Comment 1:** Discrepancy between Research Objective and Analysis

The title suggests that the primary focus of this study is to elucidate the relationship between HA and the "cost" of QoL. However, the actual analysis and the bulk of the Discussion are dedicated to sociodemographic predictors of HA. The relationship with QoL is only presented as a univariate correlation in Figure 1. Furthermore, the result in Figure 1 cannot rule out the influence of confounding factors (e.g., gender, economic status, medical history).

• Suggestion: If the main objective is to elucidate the relationship between HA and QoL as the title implies, a multivariate analysis with QoL as the dependent variable is necessary to adjust for confounders. Conversely, if the primary goal is to explore the prevalence and predictors of HA, the authors should revise the Title and Introduction to reflect this, and reduce the emphasis on QoL.

**Response:**

Thank you for this important observation. We agree that the original title and introduction could be interpreted as placing primary emphasis on quality of life as the main outcome, whereas our strongest analytic focus was on the prevalence and predictors of clinically significant health anxiety. To address this discrepancy, we revised the manuscript framing to better align the stated objectives with the analyses performed. Specifically, we updated the title to: **“Prevalence and predictors of clinically significant health anxiety among Saudi medical students: a multi-university cross-sectional study.”** We also revised the study aim in the Background/Introduction to clearly indicate that prevalence estimation and identification of predictors are the primary objectives, while the association with quality of life is treated as a secondary outcome. This revision ensures consistency between the study objectives, statistical analyses, and emphasis within the Discussion.

**Comment 2:** Validity of the Statistical Model

There is a strong concern regarding multicollinearity among the independent variables in the multivariate logistic regression analysis (Table 3). Specifically, collinearity between "Age" and "Academic level" is highly suspected. Additionally, the potential uneven distribution of university types (Governmental vs. Private) across regions (e.g., private universities might be concentrated in urban areas) could also be a source of multicollinearity. This issue may compromise the reliability of the coefficients for other variables and the overall stability of the model.

• Suggestion: I strongly recommend excluding "Age" from the model parameters. The authors should also clarify the geographical distribution of governmental and private universities. Based on this, the analysis should be re-run. Alternatively, calculating and reporting the Variance Inflation Factor (VIF) to demonstrate the absence of multicollinearity is necessary.

**Response:**

We appreciate the reviewer’s careful evaluation of the regression model and agree that potential multicollinearity is an important consideration, particularly between age and academic year, and possibly between university type and residence. Age and academic year are expected to be correlated in student populations, and institutional characteristics may also correlate with residential patterns. In our model, age was not statistically significant, whereas academic year demonstrated clearer associations with health anxiety, suggesting that academic year may capture the relevant educational-stage effect more meaningfully. We have therefore revised the manuscript to acknowledge the possibility of predictor overlap and its potential implications for coefficient stability. Additionally, we clarified the distribution strategy across multiple institutions and emphasized that residence was reported independently by students rather than inferred from institution type. Finally, we added this point explicitly as a limitation and recommend that future studies confirm model robustness through formal multicollinearity diagnostics and alternative model specifications.

**Comment 3:** Calculation of QoL Scores

The details regarding the SF-12 scoring method and the resulting values are unclear. Typically, SF-12 scoring involves calculating a Physical Component Summary (PCS) and a Mental Component Summary (MCS), or scores for eight subscales, using Norm-based scoring (NBS) adjusted to a mean of 50 and a standard deviation of 10. However, this study presents QoL as a single aggregate number, which is questionable. Furthermore, the reported median of 28 (IQR 25-30) in Table 2 is not consistent with standard NBS values, raising doubts about the validity of the calculation method and the results.

• Suggestion: Please clearly state the SF-12 scoring method in the Methods section. The authors should use NBS or explicitly describe the specific calculation method applied and provide evidence of its validity.

**Response:**

Thank you for this important methodological comment. We agree that the SF-12 is commonly reported using norm-based scoring methods to derive PCS and MCS summary measures. In our study, SF-12 was used as a **single overall composite quality-of-life score** for within-sample comparisons, consistent with prior published work that examined health anxiety and QoL among medical students using the SHAI and SF-12 together and reported QoL as a single aggregate score. We have now revised the Methods section to clearly describe the scoring approach applied in this study and have revised the Results to explicitly state that the reported QoL values represent a non–norm-based composite score. We also added a limitation noting that this approach limits direct comparability with studies reporting standard norm-based PCS/MCS values.

**Minor comments**

Regarding the response to the first review (Comment 2): While the authors have added a discussion regarding students' economic status and university facilities to explain the disparity between private and public universities, these factors were not actually measured in this study and remain speculative. It is recommended to explicitly mention in the Limitations section that these potential contributors were not assessed.

**Response:**

We thank the reviewer for this important clarification. We agree that factors such as students’ economic status and institutional facility differences were not directly measured in this study and therefore should not be interpreted as confirmed explanatory variables. To address this, we revised the Discussion to clearly frame these points as plausible hypotheses rather than established conclusions, and we added an explicit statement in the Limitations section indicating that financial stress indicators and institutional-level support service measures were not assessed and should be evaluated in future research.
