# Supplementary material for: Prevalence and predictors of clinically significant health anxiety among Saudi medical students: A multi-university cross-sectional study
Source: PLoS One. 2026 Apr 27;21(4):e0347337. doi: 10.1371/journal.pone.0347337 (PMC13120030; doi:10.1371/journal.pone.0347337)
Supplement: Attachment — Submitted filename: Reviewer Comments.docx [file pone.0347337.s004.docx]

**Reviewer Comments**

This manuscript presents interesting findings regarding the relationship between Health Anxiety (HA) and Quality of Life (QoL) among medical students. The results offer valuable insights into the association between HA and various factors such as academic year, residence, institution type, and medical/psychiatric history. In particular, the Network Analysis (Figure 3), which identifies cancer-related anxiety as a central "hub" connecting other fears, appears to be a significant and novel finding.

However, I have several concerns regarding the methodology and the presentation of the results, as detailed below.

**Major Comments**

1. Discrepancy between Research Objective and Analysis

The title suggests that the primary focus of this study is to elucidate the relationship between HA and the "cost" of QoL. However, the actual analysis and the bulk of the Discussion are dedicated to sociodemographic predictors of HA. The relationship with QoL is only presented as a univariate correlation in Figure 1. Furthermore, the result in Figure 1 cannot rule out the influence of confounding factors (e.g., gender, economic status, medical history).

- **Suggestion:** If the main objective is to elucidate the relationship between HA and QoL as the title implies, a multivariate analysis with QoL as the dependent variable is necessary to adjust for confounders. Conversely, if the primary goal is to explore the prevalence and predictors of HA, the authors should revise the Title and Introduction to reflect this, and reduce the emphasis on QoL.

2. Validity of the Statistical Model

There is a strong concern regarding multicollinearity among the independent variables in the multivariate logistic regression analysis (Table 3). Specifically, collinearity between "Age" and "Academic level" is highly suspected. Additionally, the potential uneven distribution of university types (Governmental vs. Private) across regions (e.g., private universities might be concentrated in urban areas) could also be a source of multicollinearity. This issue may compromise the reliability of the coefficients for other variables and the overall stability of the model.

- **Suggestion:** I strongly recommend excluding "Age" from the model parameters. The authors should also clarify the geographical distribution of governmental and private universities. Based on this, the analysis should be re-run. Alternatively, calculating and reporting the Variance Inflation Factor (VIF) to demonstrate the absence of multicollinearity is necessary.

3. Calculation of QoL Scores

The details regarding the SF-12 scoring method and the resulting values are unclear. Typically, SF-12 scoring involves calculating a Physical Component Summary (PCS) and a Mental Component Summary (MCS), or scores for eight subscales, using Norm-based scoring (NBS) adjusted to a mean of 50 and a standard deviation of 10. However, this study presents QoL as a single aggregate number, which is questionable. Furthermore, the reported median of 28 (IQR 25-30) in Table 2 is not consistent with standard NBS values, raising doubts about the validity of the calculation method and the results.

- **Suggestion:** Please clearly state the SF-12 scoring method in the Methods section. The authors should use NBS or explicitly describe the specific calculation method applied and provide evidence of its validity.

**Minor Comments**

Regarding the response to the first review (Comment 2):

While the authors have added a discussion regarding students' economic status and university facilities to explain the disparity between private and public universities, these factors were not actually measured in this study and remain speculative. It is recommended to explicitly mention in the Limitations section that these potential contributors were not assessed.
